# Effects of psychosocial work factors on number of pain sites: The role of sleep quality as mediator

**DOI:** 10.1186/s12891-019-2946-9

**Published:** 2019-12-11

**Authors:** J. Vleeshouwers, S. Knardahl, J. O. Christensen

**Affiliations:** Department of Work Psychology and –Physiology, the National Institute of Occupational Health, Oslo, Norway

**Keywords:** Psychosocial work factors, Sleep, Pain, Mediation, Structural equation modeling

## Abstract

**Background:**

Objective of the current study was to determine which of thirteen *specific* psychosocial work factors were related to number of musculoskeletal pain sites (NPS) prospectively over a two-year time span. Furthermore, the study aimed to explore possible mediation of these prospective relationships through sleep problems.

**Methods:**

The study was a two-wave full panel study. Participants included 6277 employees of Norwegian companies, representing a wide range of occupations. Structural equation modelling was employed to analyze direct and indirect effects of thirteen specific psychological- and social work factors on sleep problems and NPS.

**Results:**

Out of the thirteen work factors studied, positive challenges at work, role conflict, decision control, superior support, coworker support, empowering leadership, and social climate were statistically significantly related to subsequent NPS, both directly and indirectly through sleep quality. Sleep quality was related to NPS in all analyses. Most psychosocial work factors exhibited direct effects on either sleep or number of pain sites. Decision demands and control over work pacing were not statistically significantly related to sleep or pain.

**Conclusion:**

In conclusion, the results suggested sleep quality to be involved in the mechanisms by which work affects the number of pain complaints employees experience.

**Significance:**

Findings from this study suggest sleep may play a role in the complex mechanism from work stressors to musculoskeletal pain. Workplace interventions aiming to reduce musculoskeletal pain may wish to target work factors described in this study, as they affect sleep and may thereby increase number of musculoskeletal pain sites.

## Background

While the associations between single-site musculoskeletal pain and work factors have been studied for many years, multisite musculoskeletal pain (MSP) has only recently gained attention [1]. More people may be affected by multisite- than by single site pain [2], and MSP may be associated with more severe health complaints than single site pain [1]. Moreover, workers affected by multisite pain may take more sick leave [3], and may be at a greater risk of work disability [4]. The aim of the present study was to elucidate whether specific psychological and social work factors predict number of musculoskeletal pain sites (NPS), and to determine the degree to which these relationships could be explained by the impact of work factors on sleep quality.

In the present study we investigated the *number* of musculoskeletal pain sites (NPS), referring to specified parts of the body in the same timeframe. While NPS and MPS are related concepts, they are distinct since NPS includes single-site pain (i.e. NPS = 1) and differentiates between different degrees of MSP (e.g. NPS = 2 is distinguished from NPS = 5). NPS has been found to be a simple and effective way to capture pain, specifically multisite pain, and assess associated risk such as work disability [5, 6]. Counting pain locations as a measure of pain has been reported by similar occupational health studies before [5–7].

Prospective studies of effects of modifiable psychological- and social work factors on multisite musculoskeletal pain seem scarce. In their cross-sectional study of patient care workers, Sembajwe and colleagues [8] reported that high job demands, low job control, and low levels of supervisor support were associated with multisite pain. Low job satisfaction and low job control were found to predict multisite pain in a four-year prospective study of food processing company employees [1].

Most previous studies of psychosocial work factors and musculoskeletal pain, including multisite pain, have focused on factors derived from Karasek’s Demand-Control model (i.e. job demands, −control, and support) [9, 10]. In order to get a more comprehensive picture of the work-pain relationship the present study included lesser studied psychosocial work factors. While some of these work factors have been studied with other health outcomes [11–18], to our knowledge, their effects on NPS have not been studied. All psychosocial work factors included in the present study are amenable to modification and should therefore represent specific targets of employee health interventions.

*Sleep quality* has been linked to work factors as well as pain [16, 19]. Although associations between work and pain, work and sleep, and sleep and pain are established, knowledge of the underlying mechanisms by which these associations can be explained are lacking. While the experience of pain may influence sleep, the opposite is also plausible [20]. One possible way in which sleep may mediate the relationship between work factors and pain is if conditions at work evoke coping processes that spill over into the employee’s spare time causing sleep problems. Poor sleep quality may have negative health consequences and may lead to pain [21]. A second pathway is through sleep deprivation, which has been found to lower pressure-pain thresholds and increase thermal pain sensitivity [19, 22]. Sleep restriction may increase levels of Interleukin-6 which seems to be associated with pain [23].

Addressing some of the abovementioned topics, the present study examined whether aspects of sleep quality mediated relationships between psychosocial work factors and number of musculoskeletal pain sites in a large and diverse prospective sample of employees in Norway.

### Methods

### Design

This full-panel two-wave prospective study was part of “The new workplace: Work, health, and participation in the new work life” – a project carried out by the Norwegian National Institute of Occupational Health. Among other information, such as background information, coping strategies, attitudes towards work, and personality dispositions, this survey gathered data on work organization, psychological- and social work factors, and mental and somatic health complaints.

Baseline (T1) data were collected from November 2004 until November 2012. Follow-up (T2) data were collected from September 2006 until November 2014. For all participants the intervals at which data were collected was approximately two years.

### Participants

Ethical approval was obtained from the Regional Committee for Medical and Health Research Ethics (REK). Participants were recruited at the organizational level, and data included respondents from 65 different companies, spanning a wide range of different jobs. Participating organizations received results in the form of reports or presentations, which they could use to improve working conditions.

All participating employees received an information letter including a unique code to complete the survey online or a paper version of the survey with a return envelope. Participating companies were obligated to provide the possibility and time for employees to complete the questionnaire during working hours, however employees could choose to complete the survey at home.

At the time of analysis, 14,586 participants had been invited to participate at the two measurement points. Of these participants, 6277 (43.0%) completed at least one sleep item at well as at least one pain item at both baseline and follow-up, as well as answering work items at baseline. Of these participants, 44.9% were male, and 55.1% were female. Sample statistics can be found in Table 1.

**Table 1:** Sample characteristics at follow-up for employees that responded at both time points

		Employees who responded to sleep and pain items at baseline and follow-up (*N* = 6277)	
Age (y)	< 30	458	7.3%
	30–39	1586	25.3%
	40–49	2113	33.7%
	50–59	1702	27.1%
	< 59	418	6.7%
Gender	Male	2817	44.9%
	Female	3460	55.1%
Difficulties initiating sleep*	0 times	2223	35.7%
	1–3 times per month	2292	36.8%
	1–2 times per week	1044	16.8%
	3–5 times per week	485	7.8%
	6–7 times per week	182	2.9%
Disturbed sleep*	0 times	1552	24.9%
	1–3 times per month	2237	35.9%
	1–2 times per week	1279	20.5%
	3–5 times per week	826	13.3%
	6–7 times per week	337	5.4%
Neck pain*	Not troubled	5084	81.0%
	Troubled	1193	19.0%
Shoulder/Upper arm pain*	Not troubled	5197	82.8%
	Troubled	1080	17.2%
Underarm/Hand pain*	Not troubled	5781	92.1%
	Troubled	496	7.9%
Back pain*	Not troubled	5316	84.7%
	Troubled	961	15.3%
Leg pain*	Not troubled	5648	90.0%
	Troubled	629	10.0%
Number of pain sites*	0	3824	60.9%
	1	1266	20.2%
	2	679	10.8%
	3	339	5.4%
	4	127	2.0%
	5	42	0.7%

*Follow-up scores

### Exposure measures

Psychological- and social work factors were measured with the General Nordic Questionnaire for Psychological- and Social Factors at Work (QPS_Nordic_) [24]. Thirteen work factors were investigated, namely; *quantitative job demands* (four items, ρ coefficient at baseline = 0.75)*, decision demands* (three items, ρ =0.63), *positive challenges at work* (three items, ρ =0.78),), *role clarity* (three items, ρ =0.82)*, role conflict* (three items, ρ =0.70)*, decision control* (five items, ρ =0.74)*, control over work pacing* (four items, ρ =0.82), *predictability during the next month* (three items, ρ =0.62)*, support from superior* (three items, ρ =0.86)*, coworker support* (three items, ρ =0.86)*, empowering leadership* (three items, ρ =0.87)*, fair leadership* (three items, ρ =0.81), and *social climate* (three items, ρ =0.71). Items were measured with a 5-point Likert scale of frequency of occurrence ranging from “1 = very seldom or never”, to “5 = very often or always”, with the exception of *social climate* which has answer categories ranging from “1 = very little or not at all” to “5 = very much”.

### Mediator measures

Two aspects of sleep quality were measured, namely [1]; difficulties initiating sleep and [2] disturbed sleep. Since these two items reflect two distinctly separate symptoms within sleep disorder and insomnia research, and since these two sleep items showed differing results in similar studies [16], they were measured and analyzed separately [25, 26].

These two sleep quality items were measured with the following question: “Have you experienced the following symptoms in the last four weeks?” whereafter the symptoms were defined as: ‘difficulties falling asleep’ and ‘disturbed sleep’. Response alternatives included: “0 times”, “1-3 times per month”, “1-2 times per week”, “3-5 times per week”, and “6-7 times per week”. The two sleep quality items were highly correlated with a T1 correlation of ρ = 0.78 and a T2 correlation of ρ = 0.81.

### Outcome measures

NPS was measured by calculating the number of musculoskeletal pain sites. Musculoskeletal pain was measured through self-report items reflecting occurrence of ‘being troubled by’ pain in the specified body sites. ‘Being troubled by’ is a common way of describing discomfort through pain in the Norwegian language. Response alternatives included: “not troubled”, “a little troubled”, “somewhat troubled”, and “intensely troubled”.

Five separate musculoskeletal body pain sites included [1]; neck pain, [2] pain in the shoulder and/or upper arm, [3] pain in the underarm and/or hands, [4] back pain, and [5] pain in the legs. Scores on these items were dichotomized, with the cut-off point being between “a little troubled” and being “somewhat troubled” - i.e. contrasting moderate to severe pain with none or light pain - in the last four weeks. Pain items scores at follow-up were then summed to make up the count variable for number of pain sites. The resulting variable was treated as continuous in all analyses, since the statistical analyses to study mediation using a half-longitudinal mediation model rely on linear regressions. Numerical pain measures have been found to be more effective in classifying and understanding pain and pain patterns than other more complex measures, i.e. “a meaningful classification of complex pain patterns may be based on a very simple measure of pain symptoms” [27]. Furthermore, Kamaleri et al. [5] suggested that counting the number of pain sites is a simple and powerful way to measure MSP and assess associated health risks.

### Confounders

Potential confounders included in the analyses were age, sex, and skill level. Skill level was based on STYRK classifications, which is a Norwegian adaptation of the International Standard for Classification of Education (ISCED-ISCO88), reflecting number of years in education or equivalent relevant work experience. Skill level categories ranged from < 10 years of education or relevant work experience to > 16 years of education or similar work experience.

### Statistical analyses

**Fig. 1:**
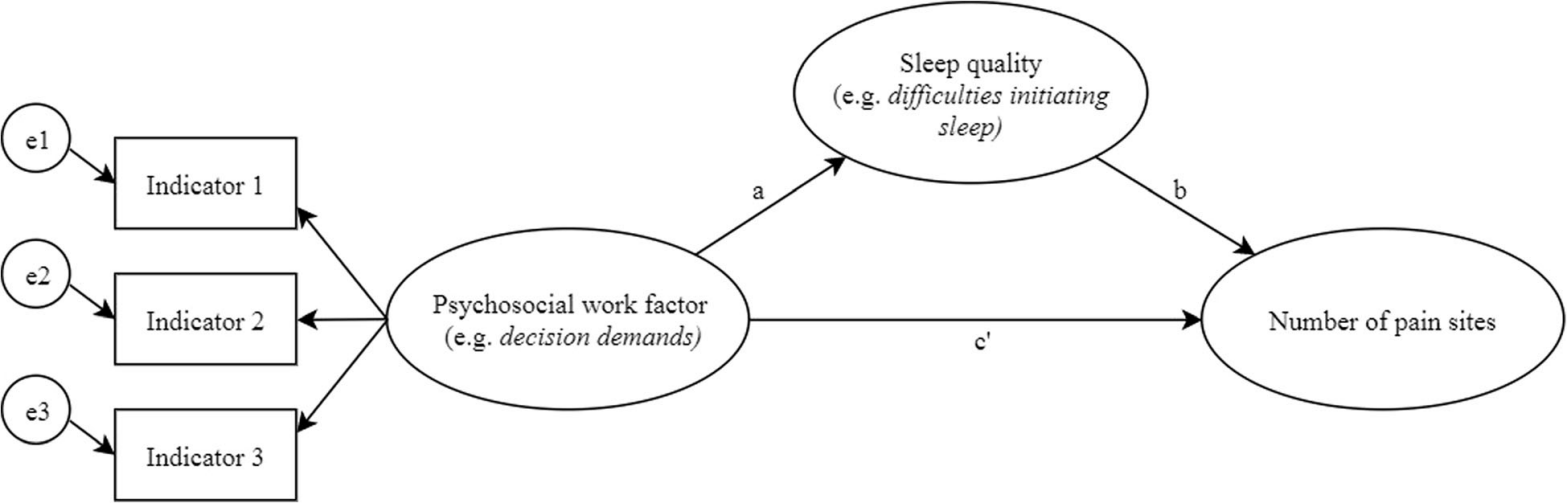
SEM model of the effect of a psychosocial work factor on multisite pain mediated by difficulties initiating sleep

All analyses were performed with MPLUS, version 7.4 [28]. Structural equation models (SEM) were run for each latent work factor variable and each sleep item separately. Since both direct as well as indirect effects were calculated in the SEM models, and since the dependent variable (NPS) was assumed continuous, MPLUS handles missing data through Full Information Maximum Likelihood (FIML) estimation. In FIML, rather than imputing the values of missing data, the value of parameters are estimated by determining the value that maximizes the likelihood function based on the sample data that is available. Parameter estimates produced via FIML are unbiased and efficient when missing data is “Missing At Random” (MAR) and multivariate normality assumptions are in place [29, 30]. An example model for one of the exposure variables and one of the mediator variables is shown in Fig. 1.

**Fig. 2:**
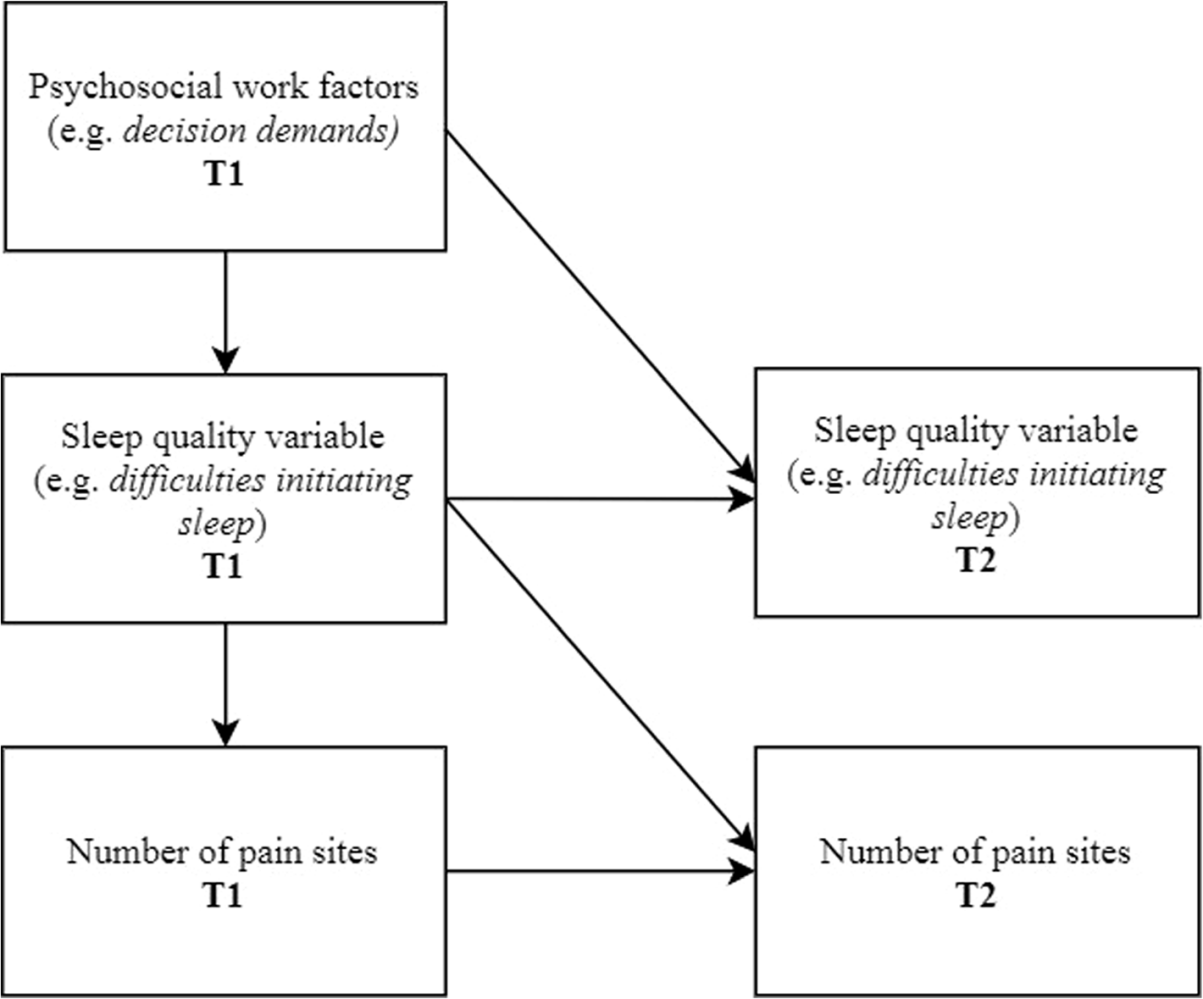
Half-longitudinal mediation model

Mediation analyses elucidate mechanisms. That is, the aim is to establish *how* an exposure causes its putative effect on the outcome [31]. In order to study mediation ideally at least three temporally separated measurement points should be included; in this case exposure at T1, mediation at T2, and outcome at T3. However, Cole and Maxwell [32] argue that *half-longitudinal mediation* may be studied in two-wave studies. In the case of half-longitudinal mediation the product of (a) the regression path of T1 exposure to T2 mediator (while controlling for T1 mediator), and (b) the regression path of T1 mediator to T2 outcome (while controlling for T1 outcome) estimates the indirect, or mediation, effect (path a x b) of exposure on outcome through the mediator [32]. A simplified graphical illustration of the half-longitudinal model is shown in Fig. 2.

Half-longitudinal mediation is based on general linear model assumptions, where both the path from exposure to mediator and the path from mediator to outcome have been calculated using linear regression. Therefore, the MSP outcome variable is treated as continuous in mediation analyses. While it is a breach of linear regression assumptions, the effect of this assumption violation is unlikely to cause problems in large datasets [33, 34], and treating a count variable as continuous may therefore be justified in the present study [35]. Furthermore, we ran zero-inflated Poisson regressions for all direct effects of the exposure and the mediator on the outcome, and no significant differences in results as compared to linear regressions were found.

Age, sex, skill level, baseline levels of corresponding sleep items, and baseline number of pain sites were included as covariates in all models. Age was categorized into the following five age groups; < 30, 30–39, 40–49, 50–59, and > 59.

Bias-corrected bootstrap confidence interval levels (BCa CI) were reported. Bootstrapping should enhance reliability when testing significance in mediation analysis [36], while also dealing with issues that may arise due to non-normality of the indirect effect [37]. One thousand re-samples were implemented in all analyses.

## Results

Neck pain was the most prevalent pain complaint, with 19% of employees experiencing moderate to severe neck pain in the last four weeks, as measured at follow-up. Underarm/hand pain was the least reported pain complaint, with 7.9% of participants reporting having experienced this pain complaint in the last four weeks. The large majority of participants reported no pain in the last four weeks (60.9%). About 20 % of participants reported pain in just one location, and 18.9% of participants reported multisite pain, i.e. pain in two or more body sites.

*Role conflict, decision control, superior support, coworker support, empowering leadership,* and *social climate* were all directly and indirectly, through both sleep quality items, related to subsequent number of pain sites. In all analyses, sleep quality statistically significantly predicted number of pain sites. Both *decision demands* and *control over work pacing* did not predict sleep or pain in any of the analyses.

Direct effects of work factors on NPS were established in at least one of the sleep quality models for most work factors. As the results show, only *decision demands* (B = -0.011, BCa CI [-0.056–0.031], *P* = 0.612), *positive challenges at work* (B = -0.043, BCa CI [-0.103–0.015], *P* = 0.153), and *control over work pacing* (B = -0.005, BCa CI [-0.057−0.048], *P* = 0.859) do not directly affect NPS. The only work factors showing differing direct effects in NPS for both sleep item models was *coworker support. Coworker support* showed statistically significant direct effects on NPS for *difficulties initiating sleep* model (B = -0.059, BCa CI [-0.107−0.001], *P* = 0.028), but not for *disturbed sleep* model (B = 0.052, BCa CI [-0.107–0.002], *P* = 0.060). Such a strong mediating effect was not found for *disturbed sleep,* although significant indirect effects exist for both sleep quality variables.

All mediation models tested showed moderately good model fit indices, with CFI’s ranging from 0.890 to 0.981, TFI’s ranging from 0.807 to 0.966. Chi-square goodness-of-fit tests were statistically significant for all models, which could be due to the large sample size. Indirect, or mediated, effects on NPS via sleep were supported for the following specific psychosocial work factors; *positive challenges at work*, *role conflict*, *decision control*, *support from superior*, *coworker support*, *empowering leadership, a*nd *social climate*. All these indirect effects were established for both mediation via *difficulties initiating sleep,* as well as *disturbed sleep.* An overview of both direct and indirect effects, including bootstrapped confidence intervals, are presented in Tables 2 and 3.

**Table 2:** Relationships between work factors and NPS (exposure at baseline and outcome at follow-up), work factors and difficulties initiating sleep (exposure at baseline and mediator at follow-up), and difficulties initiating sleep and NPS (mediator at baseline and outcome at follow-up) (*N* = 6277)

Psychosocial work factor	Work factor → Sleep quality	Work factor → Pain sites	Sleep quality → Pain sites	Indirect effect
Quantitative job demands	0.041[0.002,0.084] SE = 0.022	0.031*[0.066,0.120] SE = 0.023	0.092*[0.066,0.120] SE = 0.014	0.004[0.000,0.008] SE = 0.002
Decision demands	0.040[-0.002,0.084] SE = 0.022	0.011[-0.056,0.031] SE = 0.022	0.094*[0.067,0.120] SE = 0.014	0.004[-0.000,0.009] SE = 0.002
Positive challenges at work	-0.073*[-0.132,-0.015] SE = 0.029	0.043[-0.103,0.015] SE = 0.030	0.091*[0.065,0.119] SE = 0.014	-0.007*[-0.014,-0.002] SE = 0.003
Role clarity	0.037[-0.081,0.006] SE = 0.022	-0.053*[-0.096,-0.010] SE = 0.022	0.090*[0.064,0.118] SE = 0.014	0.003[-0.008,0.000] SE = 0.002
Role conflict	0.128*[0.076,0.180] SE = 0.027	0.117*[0.062,0.171] SE = 0.027	0.084*[0.058,0.113] SE = 0.014	0.011*[0.006,0.018] SE = 0.003
Decision control	-0.101*[-0.158,-0.034] SE = 0.032	-0.078*[-0.147,-0.010] SE = 0.035	0.090*[0.065,0.117] SE = 0.014	-0.009*[-0.016,-0.004] SE = 0.003
Control over work pacing	-0.049[-0.096,0.004] SE = 0.026	-0.005[-0.057,0.048] SE = 0.027	0.093*[0.067,0.116] SE = 0.014	-0.005[-0.010,-0.000] SE = 0.003
Predictability during the next month	-0.044[-0.104,0.007] SE = 0.029	-0.102*[-0.163,-0.047] SE = 0.030	0.090*[0.064,0.117] SE = 0.014	-0.004[-0.010,0.000] SE = 0.003
Support from superior	-0.052*[-0.084,-0.019] SE = 0.016	-0.058*[-0.090,-0.023] SE = 0.016	0.086*[0.059,0.114] SE = 0.014	-0.004*[-0.008,-0.002] SE = 0.002
Coworker support	-0.053*[-0.104,-0.011] SE = 0.024	-0.059*[-0.107,-0.001] SE = 0.027	0.090*[0.063,0.117] SE = 0.014	-0.005*[-0.011,-0.001] SE = 0.002
Empowering leadership	-0.033*[-0.063,-0.005] SE = 0.015	-0.030*[-0.060,-0.002] SE = 0.015	0.090*[0.065,0.118] SE = 0.014	-0.003*[-0.007,-0.001] SE = 0.001
Fair leadership	0.027[-0.062,0.006] SE = 0.017	-0.060*[-0.099,-0.026] SE = 0.018	0.088*[0.062,0.116] SE = 0.014	-0.002[-0.006,0.000] SE = 0.002
Social climate	-0.049*[-0.095,-0.004] SE = 0.023	-0.083*[-0.132,-0.038] SE = 0.024	0.086*[0.059,0.114] SE = 0.014	-0.004*[-0.009,-0.001] SE = 0.002

All regressions were adjusted for age, sex, skill level, and baseline levels of outcome. Values reflect Beta-estimates and confidence intervals reported are bias corrected (BCa CI)

* Significant at *P* < 0.05

**Table 3:** Relationships between work factors and NPS (exposure at baseline and outcome at follow-up), work factors and disturbed sleep (exposure at baseline and mediator at follow-up), and disturbed sleep and NPS (mediator at baseline and outcome at follow-up) (*N* = 6277)

Psychosocial work factor	Work factor → Sleep quality	Work factor → Pain sites	Sleep quality → Pain sites	Indirect effects
Quantitative job demands	0.044[-0.011,0.092] SE = 0.026	0.033[-0.009,0.075] SE = 0.022	0.072*[0.044,0.095] SE = 0.014	0.003[0.000,0.007] SE = 0.002
Decision demands	0.020[-0.031,0.074] SE = 0.026	-0.014[-0.062,0.023] SE = 0.022	0.070*[0.045,0.096] SE = 0.013	0.001[-0.002,0.006] SE = 0.002
Positive challenges at work	-0.112*[-0.177,-0.048] SE = 0.033	-0.050[-0.113,0.009] SE = 0.030	0.068*[0.043,0.093] SE = 0.013	-0.008*[-0.014,-0.003] SE = 0.003
Role clarity	-0.048[-0.103,0.000] SE = 0.026	-0.051*[-0.096,-0.007] SE = 0.023	0.066*[0.041,0.093] SE = 0.013	-0.003[-0.008,0.000] SE = 0.002
Role conflict	0.121*[0.064,0.188] SE = 0.032	0.126*[0.069,0.181] SE = 0.028	0.061*[0.037,0.088] SE = 0.013	0.007*[0.003,0.013] SE = 0.002
Decision control	-0.102*[-0.180,-0.030] SE = 0.037	-0.085*[-0.148,-0.021] SE = 0.034	0.067*[0.043,0.092] SE = 0.013	-0.007*[-0.014,-0.002] SE = 0.003
Control over work pacing	0.029[-0.038,0.090] SE = 0.031	-0.007[-0.061,0.043] SE = 0.026	0.069*[0.045,0.095] SE = 0.013	0.002[-0.002,0.007] SE = 0.002
Predictability during the next month	-0.022[-0.086,0.043] SE = 0.032	-0.105*[-0.167,-0.048] SE = 0.030	0.067*[0.042,0.093] SE = 0.013	-0.001[-0.006,0.003] SE = 0.002
Support from superior	-0.080*[-0.123,-0.045] SE = 0.019	-0.061*[-0.095,-0.030] SE = 0.017	0.063*[0.039,0.089] SE = 0.013	-0.005*[-0.009,-0.003] SE = 0.002
Coworker support	-0.070*[−0.125,-0.025] SE = 0.026	-0.052[-0.107,0.002] SE = 0.028	0.067*[0.042,0.093] SE = 0.014	-0.005*[-0.010,-0.002] SE = 0.002
Empowering leadership	-0.053*[-0.089,0.020] SE = 0.018	-0.032*[-0.061,-0.005] SE = 0.015	0.067*[0.041,0.092] SE = 0.013	-0.004*[-0.007,-0.001] SE = 0.001
Fair leadership	-0.040[-0.082,0.000] SE = 0.027	-0.062*[-0.100,-0.028] SE = 0.021	0.065*[0.040,0.091] SE = 0.013	-0.004[-0.007,0.000] SE = 0.003
Social climate	-0.061*[-0.117,-0.013] SE = 0.027	-0.091*[-0.136,-0.050] SE = 0.022	0.063*[0.039,0.089] SE = 0.013	-0.004*[-0.009,-0.001] SE = 0.002

All regressions were adjusted for age, sex, skill level, and baseline levels of outcome. Values reflect Beta-estimates and confidence intervals reported are bias corrected (BCa CI)

* Significant at P < 0.05

## Discussion

The current results suggested there are direct as well as indirect effects of psychological- and social work factors on number of pain sites (NPS), and that sleep may be one factor that contributes to explaining the complex processes linking work to pain. While effect sizes may appear small, the regression coefficients for the indirect effects represent the change in Y for every unit change in X that is mediated by M. It should be mentioned that the increase in the outcome refers to an increase in the mean number of pain sites, which could be quite meaningful even if small. So, a coefficient of 0.1 means that a unit change in the latent variable, which is probably less than one might think, depending on the variance of the latent variable, is associated with a 0.1 increase in the number of pain sites.

*Role conflict, decision control, superior support, coworker support, empowering leadership*, and *social climate* were all statistically significantly related to NPS, suggesting both direct effects and indirect effects through sleep quality. For *positive challenges at work,* direct effects on NPS were not detected, yet indirect effects through sleep quality items were established. All effects of sleep quality on NPS were statistically significant. Direct effects of the psychosocial work factors on either sleep or NPS were observed for most, but not all, work factors.

*Quantitative job demands, decision demands*, and *control over work pacing* exhibited no statistically significant effects in any analyses (see Tables 2 and 3). These work factors have, however, previously and in similar studies been associated with both sleep [16] and multisite musculoskeletal pain [8, 10]. These studies investigating effects of work on musculoskeletal pain however ranged from being cross-sectional to spanning a 5-year period. Furthermore, psychosocial work factors were operationalized differently, answer categories were dichotomized, and other covariates, such as BMI and smoking status, were included. All these differences in study methods may contribute to the discrepancies in findings. Furthermore, differences in results between previous studies and the present could be because of the fact that in the present study direct effects of these two specific work factors were tested with the SEM models. Effects may be observed in less comprehensive models. It may be the case that prospective effects of these specific work factors on multisite pain previously established became non-significant if other explanatory variables (e.g. sleep) were added to the model, suggesting the effects initially found (outside a more comprehensive model) capture other underlying mechanisms. Furthermore, previous studies have typically not conceptualized and measured the work factors as latent variables. This may have contributed to the difference in findings.

*Positive challenges at work* was found to affect NPS indirectly, both through difficulties initiating sleep as well as disturbed sleep. However, direct effects of *positive challenges at work* on NPS were not detected. This could mean that sleep quality has reduced the direct effects of this specific work factor on NPS to such an extent that is no longer statistically significant, indicating the importance of sleep in this particular sequential chain.

*Coworker support* showed statistically significant direct effects on NPS in the presence of *difficulties initiating sleep*, but not when *disturbed sleep* was included as a mediator. This could indicate that *disturbed sleep* mediates more of the effect of coworker support on NPS than *difficulties initiating sleep*, suggesting the effects of low coworker support manifest in disturbed sleep more so that in difficulties falling asleep. Nakata and colleagues investigated the effects of several work factors on insomnia, where two out of the three items comprising the insomnia scale reflected *difficulties initiating sleep* and *difficulties maintaining sleep* (which resembles *disturbed sleep).* Similar to the findings of the present study, their findings suggested that low coworker support was associated with an increased risk for *difficulties maintaining sleep,* but found no such association for *difficulties initiating sleep* [38]. While this suggest a clear difference between the two sleep quality items, and and suggests studying sleep quality items separately, it is important to note that in our results the effects of coworker support on NPS in the model with *disturbed sleep* is nearly statistically significant with a *P* = 0.06.

All direct effects of sleep quality items on NPS were statistically significant. Therefore, since indirect effects are measured by multiplying the direct paths from exposure to mediator and from mediator to outcome, the fact that some work factors generated non-significant indirect effects could be a result of a lack of statistically significant effects of the respective work factors on sleep quality. Determining which work-related stressors contribute to sleep disruptions is of importance, not only because of the discomfort of troubled sleep, but also because this appears to be a pathway to other negative health consequences, including musculoskeletal pain.

An interesting aspect of the current results was that the work factors that exhibited direct and/or indirect effects on NPS were all, bar *role conflict*, positive factors. That is, they were protective work factors with negative effects on *difficulties initiating sleep, disturbed sleep,* and *number of pain sites.* This may be of use when developing intervention programs targeting sleep- and pain problems and promoting better health in employees. While it is undoubtedly important to both reduce negative stressors as well as increase positive and supportive attitudes, this trend in effects of work on sleep and pain may indicate that protective approaches may help prevent sleep problems and pain to a greater extent.

The present prevalence rates of neck- or back pain, but also multisite pain in general seemed to be lower than in other studies [2, 39]. For instance, a 2013 report by the Norwegian Research Unit for Musculoskeletal Health (Formi) stated that 75% of Norwegians suffer some musculoskeletal complaints, with 30–50% suffering from neck pain, and half of the population reporting back pain in the last year, while 40% reported back pain in the last month [40]. This apparent discrepancy in prevalence rates was probably due to a stricter cut-off point in the current study, where pain was defined as being at least ‘somewhat troubled’ by specific pain complaints. A more appropriate comparison with the abovementioned report may therefore be the prevalence of back pain that hinders functioning which was estimated to be 11% [40]. In the current study 15.3% of respondents reported being troubled by back pain. Our findings did however not support the belief that multisite pain is more common than single site pain, as 20.2% of participants reported single site pain, while 18.9% reported pain in at least two body locations.

The present study did not investigate possible reverse causality effects of sleep and/or pain on workers’ self-reported work environment. While reverse effects may exist, the inclusion of baseline levels of sleep and pain in the models employed in the present study should attenuate their influence on results. While the present study focused on establishing direct and indirect effects of work on sleep and NPS, future studies may wish to investigate how musculoskeletal pain may affect sleep and self-reported work factors.

Defining an optimal time lag for studying the effects of work exposures and health outcomes may be challenging. Work-related strain can present itself in either physical or psychological stress effects, and these may differ in gestation time. Physical strains such as musculoskeletal pain complaints may take longer to develop [41], whereas one could argue sleep problems are more immediate and may take less time to develop. While Dormann and Griffin [42] concluded their extensive study on optimal time lags in longitudinal occupational studies by suggesting shorter rather than longer time lags, and recommending “shortudinal” designs to be used in future studies, Dormann and Zapf [43] suggest that a two year time span is required when studying stressor-strain relations.

Work has been associated with subsequent sleep problems in a number of studies. One of the few longitudinal studies investigating how work may affect sleep suggested causal relationships between job demands and sleep based on a 2-year time lag [44]. We have aimed to overcome some potential time-lag issues, as discussed before, by including baseline levels of mediator- and outcome variables as confounders.

While the results suggested that sleep problems may mediate the effects of work factors on NPS, future studies may wish to include other potential explanatory factors. For example, negative affect could play a part in explaining the relationships between work, sleep, and pain. Negative affect has been found to affect the sleep-pain pathway [45], as well as having an effect on the relationship between work factors and pain [46], suggesting it could potentially be included as an additional mediator as well as mediating the current *b* path in a more complex model. Depression may also play a part in the work-sleep-pain mechanisms. Poor psychological working conditions may contribute to depression [47], which in turn may enhance existing sleep problems and multisite pain [48, 49]. Furthermore, depression has been suggested to mediate the relationship between insomnia and multisite pain [50]. Hence, one may suspect that psychological distress mediates relationships in several places along the causal chain from work to pain, i.e. from work to sleep as well as from sleep to pain. And while the current study suported the notion of specific work factors influencing pain through sleep, the exact mechanisms remain uknown, and many possible pathways and confounding relationships are possible. For example, it is possible that a positive social climate is associated with coworker support, but that it is coworker support that has an effect on sleep and pain rather than social climate in itself. Or that other non-work related factors (e.g. marital- or relationship staturs) influence the strength of the effects of work factors on sleep and pain (e.g. the effect of coworker support on sleep). The extent of potential explanatory variables in the mechanisms exploring the path from work to sleep and pain is too vast for any single study, however exploring a set of factors at a time can contribute to the understanding of the individual relationships and their importance.

A breadth of specific factors were included in the present study. This should have implications for practice, since it offers information about specific targets of intervention. Furthermore, the inclusion of a comprehensive range of organizations, comprising a range of job types and sectors, should enhance the generalizability of the findings and render them relevant to many. Therefore, findings from the present study may be used in the construction of workplace intervention programs targeting specific psychosocial work factors, aiming to alleviate negative appraisals of these modifiable work factors, thus improving sleep and possibly reducing musculoskeletal pain due to work. Improving how employees experience these specific work factors could significantly enhance employee health and well-being, which may then aid productivity and lessen sickness absence.

## Conclusions

Findings from this study suggest sleep may play a role in the complex mechanism from work stressors to musculoskeletal pain. Workplace interventions aiming to reduce musculoskeletal pain may wish to target work factors described in this study, as they affect sleep and may thereby increase number of musculoskeletal pain sites.

## Data Availability

The corresponding author has full access to data and materials in the study. Data and materials are available upon request; please contact Prof. Dr. S. Knardahl at the National Institute of Occupational Health, Norway (contact details listed above).
